# Increased dietary 5-hydroxytryptophan reduces fearfulness in red junglefowl hens (*Gallus gallus*)

**DOI:** 10.3389/fphys.2023.1213986

**Published:** 2023-09-12

**Authors:** Kristoffer Anton Lundgren, Hanne Løvlie

**Affiliations:** IFM Biology, Department of Physics, Chemistry and Biology, Linköping University, Linköping, Sweden

**Keywords:** animal welfare, chickens, poultry, serotonin, tonic immobility

## Abstract

Our production animals typically suffer poor welfare, which can be revealed by measuring the affective state these animals are in. Negative affective state is linked to poorer welfare, and can be measured as fearfulness. While continuing to research how to improve animal welfare, a compliment to reduce negative affective state could therefore be to reduce individuals’ fearfulness, similar to how negative affective states are medicated in humans. A proposed mechanism for this is via the monoaminergic systems. This is based on previous studies across species that have linked the serotonergic system and fear-related behaviour. We here aimed to experimentally manipulate the serotonergic system in red junglefowl hens (*Gallus gallus*), the main ancestor of all domesticated chickens. We measured fearfulness as latency remaining immobile in a tonic immobility test, and did so both before and after our experimental manipulation. We set out to experimentally manipulate the serotonergic system via sub-chronic dietary treatment of 5-hydroxytryptophan (the precursor to serotonin). Our dietary manipulation of 5-hydroxytryptophan significantly reduced measured fearfulness in the manipulated hens, while latency in tonic immobility did not significantly change in our unmanipulated, control hens. This finding is promising since it indicates that increased tryptophan levels can be used to reduce fearfulness. Additionally, our result suggests that this can be done non-invasively via food (instead of injections), thus presenting a potentially feasible manipulation also for larger settings. Nevertheless, the serotonergic system is complex and its role in modulating behaviour in the fowl should be explored further to evaluate our findings, and more directly explored also in a production setting.

## 1 Introduction

Animals across a range of species typically suffer poor welfare in an industrial setting. Poor welfare can be measured by estimating the affective state (i.e., emotion, mood, Mendl et al., 2010) of individuals, where individuals with poorer welfare often are in a more negative affective state (e.g., Harding et al., 2004; Forkman et al., 2007). For example, negative affective state measured as fearfulness (Jones, 1996; Forkman et al., 2007) increased when animals are housed in poorer conditions (e.g., Hansen et al., 1993), and is linked to higher stress hormone levels (e.g., de Haas et al., 2012). Further, being in a negative affective state can enhance negative impacts of poor welfare (Jones, 1996). Despite that animal welfare research focuses on improving animal welfare, the problems remain (Nicol, 2020). Therefore, while continuing to work on external factors that can improve animal welfare, underlying mechanisms which in turn can improve the affective state of animals, should also be explored. This route is very commonly used to improve human affective state, when medicating and reducing depression and anxiety (e.g., Hamon and Blier, 2013). Such an approach can also be successfully used to alter affective state in animals (e.g., Hymel and Sufka, 2012; reviewed by Neville et al., 2020).

Monoamines (i.e., neurotransmitters) have broadly, and across species, repeatedly been shown to affect a range of behaviour. Focusing on serotonin, this monoamine can directly or indirectly affect behaviour of relevance to animal welfare (e.g., Coppens et al., 2010; Stracke et al., 2017; Abbey-Lee et al., 2018; 2019), and experimental alteration of serotonin has successfully reduced fearfulness or related behaviours (e.g., several fish species, Winberg and Thörnqvist, 2016; mice, *Mus musculus*; Foltran et al., 2020; Mediterranean field crickets, *Gryllus bimaculatus*; Lundgren et al., 2021). Hence, due to the conserved nature of the monoaminergic systems (Gunnarsson et al., 2008), manipulation of the serotonergic system may alter fearfulness also in agricultural species.

We here focus on monoaminergic manipulation of fearfulness in chickens. The domestic chicken (*Gallus gallus domesticus*) is today the most numerous bird in the world and one of our most intensely farmed animals, with billions raised yearly for meat and egg production (reviewed by, e.g., Nicol, 2015; Pizzari, 2016). These chickens suffer from a variety of severe welfare problems, such as cannibalism, feather- and vent pecking (Lambton et al., 2015; Nicol, 2015). To measure their welfare, estimating level of fearfulness is commonly used to describe their negative affective state (Forkman et al., 2007; Laurence et al., 2012). Also in chickens is the serotonergic system linked to a range of behaviour (e.g., Shea et al., 1991; Dennis et al., 2013; de Haas and van der Eijk, 2018; Birkl et al., 2019), including fear-related behaviour (e.g., Hicks et al., 1975; Hennig, 1980; Phi Van et al., 2018). Therefore, with the extended aim to improve their welfare, we specifically investigate if experimental manipulation aimed to target the serotonergic system could reduce fearfulness in chickens.

Previous work on manipulations of the serotonergic system in both fowl and other species have primarily been done via drug injection (e.g., Hennig, 1980; Foltran et al., 2020; Lundgren et al., 2021) or through drinking water (Dulawa et al., 2004). Both of these approaches have their drawbacks: while injection is fast and the amount of drug given is precise, injections can cause stress to test subjects (Morton et al., 2001), may need repeated injections to keep stable levels (Hennig, 1980), and is thus impractical to large number of individuals. Administering through the drinking water has minimal stress effect on the test subject and is practical for larger number of individuals, but dosing of the drug and time of dosing will be less precise. Considering these drawbacks, we opted for an alternative approach in administering the drug in a non-invasive way, by manipulating the food given.

We chose to focus on alteration of 5-hydroxytryptophan levels (5-HTP), since this is the precursor of serotonin (where the amino acid tryptophan is metabolised into 5-HTP, which quickly is metabolised into serotonin, e.g., O'Mahony et al., 2015). We did so by experimentally manipulating the amount of 5-HTP given in feed. Increase of dietary tryptophan has resulted in higher plasma and turnover levels of serotonin in the brain of chickens (e.g., van Hierden et al., 2004b) and other animals (e.g., Winberg et al., 2001). Alteration of tryptophan in domestic fowl has previously shown to affect behaviour, such as feather pecking (Birkl et al., 2019) and aggression (Shea et al., 1991). Using a dietary manipulation allowed us to control the amount of drug each individual was given, whilst not having potentially stressful effects of injections. However, chickens have the ability to store food in their crop (Svihus, 2014), and may therefore not digest the manipulated food immediately. Thus, drug administration via food has a similar drawback as administration through water in that the timing of the dose will be less precise. As a way to get around this, we chose to study the sub-chronic effect (i.e., longer than acute, but shorter than chronic, Dulawa et al., 2004) and dose our chickens for several days in sequence.

We used this dietary manipulation of the serotonergic system on red junglefowl (*Gallus gallus*) hens. The red junglefowl is a growing model species for research on animal behaviour and animal welfare, and is behaviourally and cognitively similar to its descendant the domesticated chicken (reviewed by Garnham and Løvlie, 2018). Further, the red junglefowl is the main common ancestor of all domestic chickens (Fumihito et al., 1994), which should enable our findings to be general for chickens broadly and not limited to certain strains of fowl (e.g., broilers, layers).

## 2 Materials and methods

### 2.1 Animals and housing

In October and November 2021, red junglefowl hens (*n* = 48) from a larger, pedigree-bred population maintained at Linköping University (see Sorato et al., 2018 for further details) were used for this study. These hens came from 23 parental pairs (where none were half-siblings), with 1–4 from each parental pair. All hens from the cohort were used, and these were raised and housed together throughout life. Hens took part in the study between 55 and 58 weeks of age (i.e., when sexually mature). To facilitate identification, all hens were wing-tagged as chicks with unique numbers. Hens were divided up in two groups: treatment (*n* = 24) and control (*n* = 24), with chickens from families with multiple offspring represented in both groups. During this experiment, hens were housed together with roosters in female-male pairs in enclosures (60 cm × 45 cm × 50 cm, L x W x H) containing a perch, shelter, sawdust, and a laying/brooding area, light (6:30 a.m. to 6:30 p.m.) and with *ad libitum* access to commercial poultry feed and water. The experiment was carried out in accordance with Swedish ethical requirements (Linköping Ethical Committee, ethical permit number 288-2019).

### 2.2 Experimental set-up

All hens were tested individually and had previously taken part in behavioural studies and were used to human presence and handling (Rubene and Løvlie, 2021; Garnham et al., 2022a). During testing, all hens were exposed to a tonic immobility test (see below) twice: once on the day before the experimental dietary manipulation began and once on the day after the final dose (i.e., 5 days later).

### 2.3 Experimental dietary manipulation

Based on previous work (Donato et al., 2015), our experimental manipulation used 5-hydroxytryptophan (5-HTP, Sigma-Aldrich), the precursor to serotonin. 5-HTP was diluted in Phosphate buffered saline (PBS, Sigma-Aldrich), and mixed together with cottage cheese at the time of dosing. Cottage cheese was used as it is an attractive food source for the hens, and it was easily mixed with the 5-HTP-mixture. One day prior to testing, each hen was weighed (to the nearest gram) and the dosage of 5-HTP received was calculated to 30 mg of 5-HTP per kg of body weight (based on Donato et al., 2015). Our females weighed between 722 and 1,103 g, with the average weight of 877 g. This resulted in doses ranging between 21.66 and 33.09 mg, with the average dose of 26.43 mg for our hens. Twenty-four hens were individually fed 7 g cottage cheese mixed with the drug for 5 consecutive days at around 4 p.m. Control hens (*n* = 24) were given the same handling as above, but were fed cottage cheese (7 g) mixed with only PBS. All females ate all cottage cheese given to them, on all 5 days. Due to logistical constraints, we were here only able to use one dose of 5-HTP for our treatment.

### 2.4 Measuring fearfulness

Hens were individually exposed to a tonic immobility test, a test commonly used in poultry research to measures fearfulness, where longer latency immobile describes more fearful individuals (Gallup, 1979; Hennig, 1980; Jones, 1986; Forkman et al., 2007). Each hen was tested by the same observer (KL) to minimize any differences in handling. We followed the protocol routinely used by our group (e.g., Favati et al., 2015; Zidar et al., 2017; Garnham et al., 2019). To induce tonic immobility, the observer placed the hen on her back in a cradle and gently held her down with one hand over the chest and the other hand over her head, whilst avoiding eye contact with the hen. After 15 s, the observer lifted his hands and tonic immobility was considered induced if the hen remained on her back for at least 3 s. Fearfulness was measured as the time (in seconds) a hen took until she stood upright again after tonic immobility had been induced, with longer latency used as a measure of higher level of fearfulness (Forkman et al., 2007). If a hen did not enter tonic immobility after a maximum of three attempts to induce this, she was given a tonic immobility latency of 0 s (this only happened to one hen prior to treatment, and eight after). Hens that did not come out of tonic immobility within 600 s were given a latency of 600 s (this only happened to four hens prior to treatment, and three hens after).

### 2.5 Statistical analyses

R version 4.0.1 (R Core team, 2020) was used for statistical analyses. The measure of fearfulness (i.e., latency to stand, in seconds) did not follow the assumptions needed for parametric statistics. Therefore, non-parametric statistical tests were used. To test for any unintended differences between later treatment and control hens, a Mann-Whitney U test was used by comparing fearfulness of our 5-HTP treated hens and control hens (i.e., comparing data from two independent samples), prior to the experimental manipulation was carried out. To test for the effect of our 5-HTP manipulation, two separate Wilcoxon matched pair tests were used to compare fearfulness of the same individual between first and second trial of the tonic immobility test (hence for hens in the treatment group: before and after manipulation) for hens in the treatment and control group, separately. Further, the difference in fearfulness between first and second trial was compared between hens in the treatment and control groups, by running a generalized liner model with Poisson distribution with ∆fearfulness (change in latency to stand per hen over the two trials, calculated as latency in first test occasion minus latency in second test occasion) as the response variable with type of treatment (5-HTP vs. control; categorical variable) as a fixed effect. To fit model assumptions, ∆fearfulness was transformed according to the formula: transformed = ∆fearfulness + 491 + 1, as some values were negative (i.e., when a hen increased her latency to stand). Transformation of ∆fearfulness resulted in that the lowest value was 1. The statistical significance of the fixed effect was assessed based on a 95% credible interval (CI) around the mean (β), and considered to be significant when the 95% CIs did not overlap zero.

## 3 Results

**FIGURE 1:**
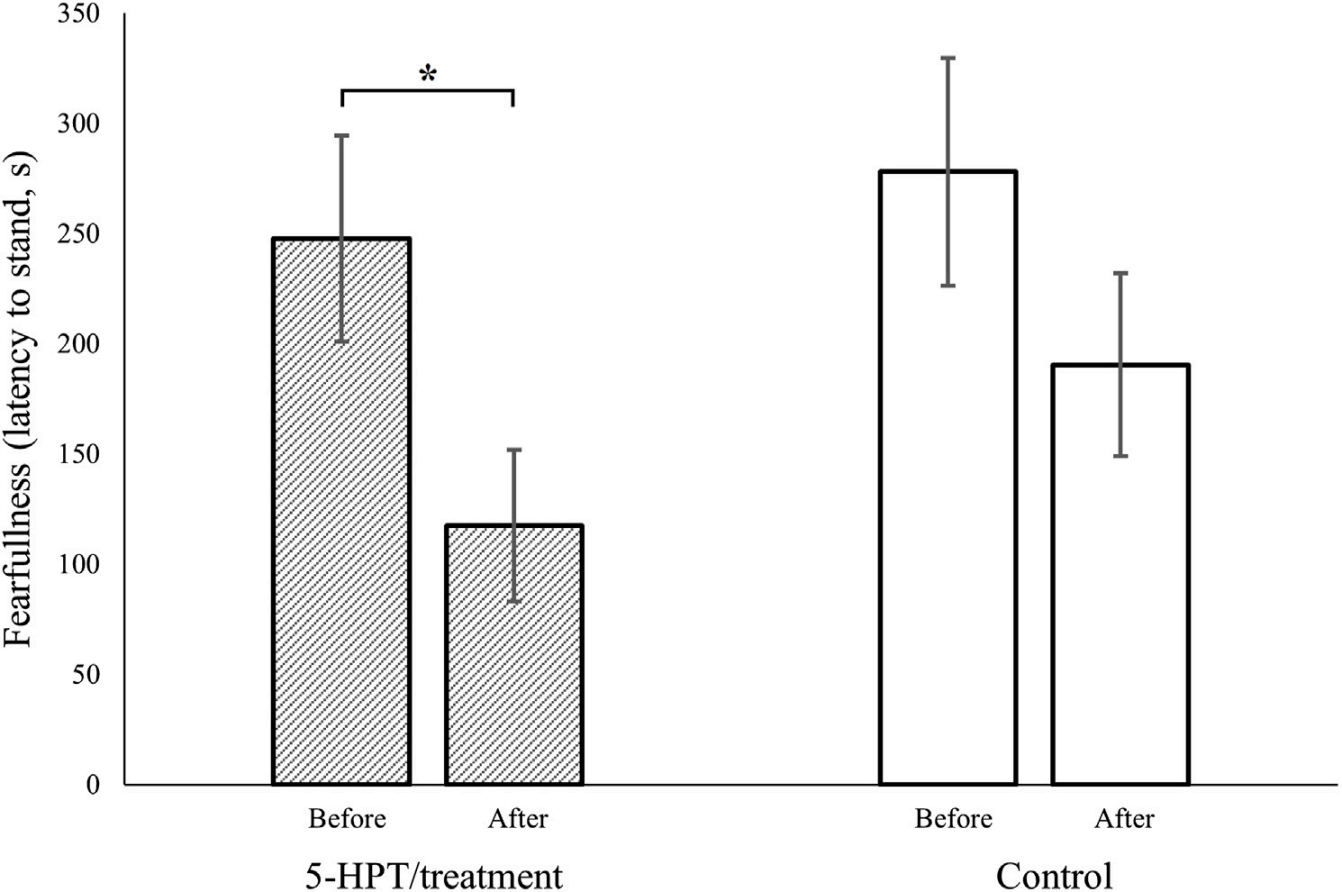
Fearfulness (latency to stand, in seconds, after induction of tonic immobility) in red junglefowl hens was reduced after intended serotonin manipulation (via increased dietary 5-HTP, 5-hydroxytryptophan, the precursor to serotonin, dashed columns), while no such significant reduction was observed in our control hens (empty columns). Before vs. After refer to first and second trial in a tonic immobility test, respectively. Columns present mean, bars present SEM, * symbolizes *p* ˂ 0.05, and that the 95% credible intervals did not overlap zero.

Prior to our experimental manipulation, there was no significant difference in fearfulness between the later 5-HTP-treated hens and our control hens (5-HTP-treated: *n* = 24, mean ± SE = 247.71 ± 46.71; control: *n* = 24, mean ± SE = 277.91 ± 51.67, W = 321.5, *p* = 0.49; Figure 1).

However, our 5-HTP manipulation significantly lowered fearfulness in the 5-HTP-treated hens (before manipulation: *n* = 24, mean ± SE = 247.71 ± 46.71; after manipulation: *n* = 24, mean ± SE = 117.46 ± 34.28, W = 386.5, *p* = 0.04, Figure 1), while no such significant decrease was observed in our control hens (first test occasion: *n* = 24, mean ± SE = 277.91 ± 51.67; second test occasion: *n* = 24, mean ± SE = 190.37 ± 41.44, W = 343.5, *p* = 0.25, Figure 1). Further, this reduction in fearfulness was confirmed to only be significant in our 5-HTP manipulated hens, by our generalized linear model (∆fearfulness, 5-HTP-treated: *n* = 24, mean ± SE = 130.25 ± 43.18; ∆fearfulness, control: *n* = 24, mean ± SE = 87.54 ± 52.84; *β* = −0.07, 95% CIs = −0.09, −0.05).

## 4 Discussion

We set out to test if fearfulness could be reduced via dietary manipulation of the underlying monoaminergic systems, specifically focusing on the serotonergic system. We did this by enhancing food given to individual red junglefowl hens with 5-hydroxytryptophan, for 5 consecutive days (i.e., we did a sub-chronical manipulation). We here show that this relatively short feed manipulation reduced the latency treated hens remained in tonic immobility, a common measure of fearfulness in poultry (where shorter latencies implies less fearful individuals). In our control hens were fearfulness also somewhat reduced in the second test, although not as strongly as what we observed for our experimental hens, and not significantly. The observed reduction in fearfulness in our control hens therefore confirms that habituation to the test can reduce latencies in immobility (e.g., Ratner and Thompson, 1960), and that the effect we observed in our experimental birds was mainly explained by our dietary manipulation.

We used 5-hydroxytryptophan to experimental manipulate feed given to our treatment hens. 5-hydroxytryptophan is a metabolite of tryptophan, which in turn is metabolised rapidly into serotonin (O'Mahony et al., 2015). Our finding that a manipulation set out to affect the serotonergic system affected behavioural responses, is confirmed across taxa (e.g., several fish species, Winberg and Thörnqvist, 2016; mice; Foltran et al., 2020; Mediterranean field crickets; Lundgren et al., 2021). When focusing on previous manipulation of the serotonergic system affecting fear-related behaviours, that is also confirmed across vertebrate species (e.g., humans, Hindi Attar et al., 2012; chickens; Nicol, 2015; mice; Waider et al., 2019). These findings seem to be general, independent of method of manipulation. For example, in mice, manipulated serotonin levels via injection or diet gave similar reduction in levels of brain serotonin (Foltran et al., 2020). It is promising that the use of a non-invasive method produces similar results to also more precise, invasive methods, as this should ease translation of the method to a more industrial setting.

Tryptophan is an essential amino acid obtained only through food. Poultry feed already have some percentage tryptophan (e.g., van Hierden et al., 2004b; Barua et al., 2021), and this percentage can relatively easily be enhanced further (e.g., Winberg et al., 2001), thus should be possible to increase commercially. However, there are several aspects that should be clarified before this commercial step can be taken. Firstly, it should be confirmed that dietary manipulation of 5-HTP indeed increased serotonin levels in treated hens (as shown previously in chickens, van Hierden et al., 2004b), and whether this resulted in increased peripheral serotonin levels (in plasma), and/or in the central nervous system (brain). The vast majority of serotonin (ca 95%) is synthesised in the gut and circulated in the blood (see reference in, e.g., Yan et al., 2018). The synthetic cascade is similar in the gut as in the brain (tryptophan—5-hydroxytryptophan–serotonin). However, since peripheral serotonin cannot cross the brain-blood barrier (Mann et al., 1992), serotonin synthesis in the gut and in the brain has two separate pathways and the function of serotonin can differ in the two separate networks. Across vertebrates, serotonin in both networks can affect aspects of relevance to welfare (O'Mahony et al., 2015), and to poultry welfare more specifically (e.g., feather pecking, van Hierden et al., 2004a; van Hierden et al., 2004b; de Haas and van der Eijk, 2018). Yet, the role of serotonin does not need to be parallel in plasma and brain. For example, peripheral serotonin levels were unaltered, while serotonin levels in the brain were affected by comparing broilers given probiotic diet vs. control birds (Yan et al., 2018). How and why our dietary manipulation affected behaviour, is currently unknown, and investigation of whether plasma or brain serotonin levels changed could give hint to the underlying mechanism through which our dietary manipulation acted. Further, even if the underlying mechanism to observed behavioural alteration is known, further work needs to consider that serotonin manipulation via food will not result in individual dosing. Thus, both the preferred dose, and the acceptable span of levels individuals obtained dependent on differential food intake, must be explored further. This is because dose response curves of serotonin and responses to the same fearfulness test we here used, the tonic immobility test, shows that doses can produce quite varying and non-linear responses (Hennig, 1980). In addition, alteration of feed can cause amino acid imbalances. Further, high levels of serotonin can cause serotonin syndrome in which a range of abnormal behaviours can be observed, such as head weaving, hyperactivity and twitching (reviewed by, e.g., Haberzettl et al., 2013).

On a slightly different note, yet of relevance for animal welfare, manipulation of serotonin levels in domestic fowl can reduce feather pecking (e.g., van Hierden et al., 2004a; de Haas and van der Eijk, 2018). Feather pecking is a large welfare problem in the poultry industry, and serotonin manipulations can reduce certain aspects of feather pecking (van Hierden et al., 2004b). Therefore, further work on how to enhance serotonin levels could have positive influences on chicken welfare for multiple reasons. However, the underlying mechanisms for fearfulness and feather pecking are still not fully understood, and warrant future research.

We here used a well-established behavioural test of fearfulness commonly used on poultry (not only chickens, but, e.g., quail, Jones et al., 1991). Further, the use of latency to come out of immobility after induction of tonic immobility is used as a measure of fearfulness also across other animals (reviewed by, e.g., Hennig, 1980). Yet, this measure does not always correlate with other aspects of fearfulness, or positive welfare. Longer latencies in tonic immobility has been linked with other measures of fearfulness (reviewed by, e.g., Forkman et al., 2007), but also with stress (Zulkifli et al., 1998; Kozak et al., 2019). Thus, what exactly the tonic immobility test is measuring is still debated (reviewed by, e.g., Humphreys and Ruxton, 2018). Nevertheless, the test seems to capture negative affective state. We recently showed, in the same population of junglefowl here used, that latency to remain in tonic immobility was moderately positively correlated with responses to ambiguous, intermediate cues in a cognitive judgement bias test (Garnham et al., 2022b). A judgement bias test is widely used as one of the very few confirmed methods to measure positive affective state of animals (reviewd by, e.g., Lagisz et al., 2020; Neville et al., 2020). That a low fearfulness correlated with a higher positive affective state is promising in that what we here have shown may translate to not only “less bad welfare,” but also improved welfare. However, this should be directly tested by manipulation of the serotonergic system and measure positive affective state, suggestively by the use of a judgement bias test. Suggesting such a link is also our previous findings that responses to a judgement bias test is linked to variation in the monoaminergic systems (Zidar et al., 2018; Boddington et al., 2020). In the same population as here used, we previously shown that brain gene expression of the DRD1 (dopamine receptor D1, another important neurotransmitter of the monoaminergic systems) gene, was positively correlated with positive affective state measured in a judgement bias test (Boddington et al., 2020). In layer hens, brain turnover rate of dopamine was positively correlated with positive affective state measured in a judgement bias test (Zidar et al., 2018). On the other hand, in a smaller sample of individuals from the same population of red junglefowl here used, no correlation was observed between brain gene expression of a range of serotonergic genes and latency remaining in tonic immobility (Lundgren et al., 2021). Thus, the relationship between negative and positive affective state, and its underlying mechanisms have to be explored further.

Clarification of the link between responses to tonic immobility and measured positive affective state in a judgement bias test is relevant both as it may establish how negative and positive affective states are linked, but also if tonic immobility test can be used instead of judgement bias tests. Judgement bias tests are both time consuming and take a lot of handling of the animals, as the values of negative and positive cues need to be learned (see, e.g., Zidar et al., 2018; Garnham et al., 2019). The use of a tonic immobility test is thus more time efficient, although this too needs individual handling and testing of birds. When the relationship between dietary manipulation of underlying monoaminergic systems is better understood, and if reduction in feather pecking is produced as a side-effect of serotonin enhancement, visual inspection of flocks with regard to feather pecking could perhaps act as a group-level measure of altered affective state. However, this is currently only a speculation.

Overall, the relationship between negative and positive affective states in chickens and other animals needs to be better described. We recently showed that the relationship between responses to tonic immobility and responses in the judgement bias test depends on whether chicks or adults were tested (e.g., the relationship was only found in chicks, and not in adults, Garnham et al., 2022b). This suggests that before generalizing to different ages and strains of fowl, the generality of our findings should be investigated further since our results may differ for broilers (i.e., chicks) vs. layers (i.e., adults). Further, as a first step, we here only tested females. In our population, we have previously observed no or weak effects of sex on responses to the tonic immobility test (e.g., Favati et al., 2015; Zidar et al., 2017; Lundgren et al., 2021; Garnham et al., 2022b). However, sex effects in responses to monoamine manipulation on behaviour also warrant future exploration.

## 5 Conclusion

We have here shown that a relatively short (5 days) experimental manipulation of diet with the aim to alter the serotonergic system of red junglefowl hens, the main ancestor of all domesticated chickens, reduced how fearful these were, measured in a tonic immobility test. Our manipulation was non-invasive through 5-hydroxytryptophan enhanced food given to the birds. Tryptophan, the precursor of serotonin (and from which 5-hydroxytryptophan in turn is metabolised), is already part of commercial chicken feed. Although still in its early stage, these aspects together produce promising results for a hopefully practical method to reduce fearfulness of also industrial birds, which in turn may improve their animal welfare. Future work should evaluate tryptophan enhanced food in industrial settings, together with the drug dosage needed to produce satisfactory levels of animal welfare.

## Data Availability

The original contributions presented in the study are included in the article/Supplementary Material, further inquiries can be directed to the corresponding author.
